# The Role of Long Non-Coding RNAs in Ovarian Cancer Cells

**DOI:** 10.3390/ijms25189922

**Published:** 2024-09-14

**Authors:** Anna Golara, Mateusz Kozłowski, Aneta Cymbaluk-Płoska

**Affiliations:** Department of Reconstructive Surgery and Gynecological Oncology, Pomeranian Medical University in Szczecin, Al. Powstańców Wielkopolskich 72, 70-111 Szczecin, Poland; anka39143@gmail.com (A.G.); mtkoozo@gmail.com (M.K.)

**Keywords:** long non-coding RNA, ovarian cancer, NEAT 1, SOX21-AS1, H19, LOXL1-AS1, MAFG-AS1, GAS5, LINC0015, pseudogenes

## Abstract

Among the most deadly malignancies that strike women worldwide, ovarian cancer is still one of the most common. The primary factor affecting a patient’s survival is early lesion discovery. Unfortunately, because ovarian cancer is a sneaky illness that usually manifests as nonspecific symptoms only in advanced stages, its early detection and screening are challenging. A lot of research is being conducted on effective methods of diagnosing and treating ovarian cancer. Recently, non-coding RNAs (ncRNAs) have gained great popularity, which are considered to be the main regulators of many cellular processes, especially those occurring in cancer. LncRNAs are also being studied for their therapeutic use in the treatment of ovarian cancer and their use in diagnostics and as indicators of poor prognosis. In this article, we reviewed lncRNAs described in the literature that may play an important role in ovarian cancer.

## 1. Introduction

The term “ovarian cancer” (OC) refers to a wide spectrum of cancers developing from cells of the ovary, fallopian tube or peritoneum, with various histological, clinicopathological origins and molecular features [1]. Epithelial ovarian cancer is a highly variable illness that may be classified into five primary histotypes based on genetic differences and clinical characteristics [2]. One of the deadliest malignancies to affect women globally is still ovarian cancer [3], whose survival rates are mostly reliant on early identification [4]. The sneaky nature of ovarian cancer makes its early identification and screening hard [5]. Patients typically have non-specific symptoms, which are noted in advanced disease [6]. The most common symptom reported by patients with high-risk epithelial ovarian cancer is abdominal or pelvic pain. As the size of the tumor increases, so do the proportion of symptomatic women and the quantity of symptoms [7]. Ovarian epithelial tumor indicators, or cancer antigen 125 (*CA125*) and human epididymis protein 4 (*HE4*), are helpful in diagnosing, tracking efficacy, and keeping an eye out for relapses. However, the cancer stage, histological type, age, and menopausal state can also affect the levels of these markers. Additionally, they can occasionally be raised in a number of benign and malignant disorders affecting the female reproductive system. Although carbohydrate antigen 19-9 (*CA199*) and carcinoembryonic antigen (*CEA*) can also be raised, they are not specific to OC as they are frequently elevated in gastrointestinal cancers. Although ovarian tumors can be identified by imaging, a precise diagnosis and differentiation of benign from malignant lesions cannot be made. Imaging tests are also unable to detect an alteration until it has grown to a sufficient size [8]. On the other hand, multigene tests can be utilized to detect breast cancer susceptibility gene 1 (*BRCA1)* and breast cancer susceptibility gene 2 (*BRCA2)* mutation carriers and individuals at high risk of OC, which makes it easier to select therapeutic drugs and estimate their prognosis [9]. 

Surgery, radiation, targeted therapy, hormone therapy, immunological therapy, and polyadenosine diphosphate ribose polymerase inhibitor maintenance therapy are all used to treat OC patients [10,11]. Most patients with ovarian cancer experience clinical remission following their initial therapy; nevertheless, 70% of patients return and rapidly become resistant to platinum, and the five-year survival rate is only 46% [12]. Although there has been progress in the treatment of ovarian cancer, the disease is still lethal. Of the histological types, high-grade serous ovarian cancer (HGSOC) is the most frequent (>80%). Five-year survival rates for ovarian cancer patients are 13% and 27%, respectively, with approximately 80% of patients presenting in advanced stages III and IV [13,14].

**Figure 1:**
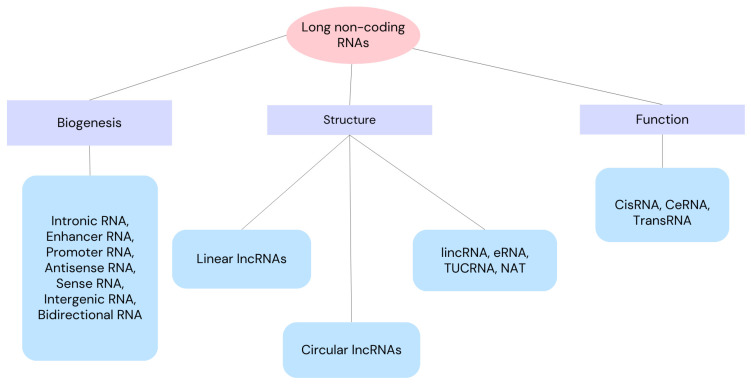
Classification of long non-coding RNAs into classes and subclasses according to their function, biogenesis and structure. lincRNA—long intergenic non-coding RNA, eRNA—enhancer RNA, TUCRNA—transcribed ultra-conserved RNA, NAT—natural antisense transcript, CisRNA—cis-acting RNA, CeRNA—competitive endogenous RNA, TransRNA—trans-acting RNA.

Non-coding RNAs (ncRNAs) are regarded as master regulators of a number of cellular processes, including ferroptosis’ molecular regulation. As a result, ncRNA-based therapies may be a good option for cancer treatment [15]. Small non-coding RNA (sncRNA) has fewer than 200 nucleotides, while long non-coding RNA (lncRNA) has more than 200 nucleotides. Both types of ncRNA have the capacity to encode proteins, either fully or partially [16,17,18]. Since lncRNA is important for translation, post-transcriptional transcription, gene transcription, and epigenetic modification [19], aberrant expression or lncRNA dysfunction can result in a number of disorders [20]. We can divide lncRNAs according to their function, biogenesis and structure as depicted in Figure 1. Through a variety of manners, lncRNAs can affect cell proliferation, apoptosis, migration, and invasion, which can impact the development of tumors [21,22]. Through the recruitment of regulatory components to the locus and the regulation of their functions, lncRNAs can operate as sponges, absorbing micro-RNAs (miRNAs) or regulating surrounding genes in cis. Additionally, proteins can be bound by lncRNAs to enhance their stability, function, and avoid destruction. Thus, lncRNA–protein interactions are crucial for the development of cancer and its spread [23,24,25]. In this review, we present an up-to-date literature review as of September 2024, including clinical trials. The purpose of this review is to determine the role of long non-coding RNAs in ovarian cancer cells. Here, we review NEAT 1, SOX21-AS1, H19, 74 LOXL1-AS1, MAFG-AS1, GAS5 and LINC0015 long non-coding RNAs because of their involvement in ovarian cancers.

## 2. Long Non-Coding RNA NEAT1

*NEAT1*, or nuclear-enriched autosomal transcript 1, is involved in the carcinogenesis of cancer [26]. Due to its potential involvement in the beginning and advancement of cancer, as well as the fact that its aberrant expression is associated with clinical aspects such as metastasis, treatment resistance, and patient survival, *NEAT1* exhibits characteristics of a tumor driver [27,28]. In a variety of cancers, *NEAT1* can enhance stem cell characteristics and mediate an oncogenic phenotype [29]. By focusing on different miRNAs, RNA-binding proteins, mature mRNAs, and non-protein-coding genes, lncRNAs control epithelial–mesenchymal transition (EMT) processes and tumor spread in malignancies of the reproductive system [30]. 

A high expression of *NEAT1* is strongly connected with distant metastases, tumor stage, and a poor prognosis in ovarian cancer when considered as an independent factor [31]. *NEAT1* promotes ovarian cancer cells’ EMT, invasion, and migration by mediating the expression of tight junction protein three (*TJP3*) and blocking the activity of miR-1321 [30]. *NEAT1* increases the growth and invasion of cancer cells via binding to the human antigen R (*HuR*) protein and sponging *miR-124-3p*. HuR, a particular RNA-binding protein (RBP) plays important role in stabilizing and modulating the translation of many of its target mRNAs including *p21*, *c-fos*, *VEGF*, *MKP-1*, *TNF-α*, *Bcl-2*, *Mcl-1*, and *p53.* Higher *HuR* mRNA levels are also positively correlated with the International Federation of Gynecology and Obstetrics (FIGO) tumor stage and the occurrence of lymph node metastasis [32]. In individuals with ovarian cancer, high *NEAT1* expression is associated with a worse prognosis and a lower survival rate. The expression of Rho-related protein kinase 1 (ROCK1), a gene linked to metastasis, can be targeted by *NEAT1* to accelerate ovarian cell invasion and migration. Consequently, *miR-382-3p* inhibited the spread of tumors by focusing on ROCK1’s 3′-UTR [33].

By blocking caspase-3 action, *NEAT1* overexpression can also promote cell division and decrease apoptosis in ovarian cancer cells. *NEAT1* has the ability to upregulate *miR-34a-5p* and induce the expression of B-cell lymphoma-2 (BCL2). Elevated *NEAT1* expression can also speed up a cell’s entry into the S phase and shorten the G0/G1 phase. Consequently, *NEAT1* knockdown enhances apoptosis and decreases cell growth [34]. Abnormal *NEAT1* expression was discovered in HGSOC by Yong et al. [35]. As an oncogene, *LIN28B* can bind to *NEAT1* and stabilize its expression. This means that *NEAT1* can control the growth, proliferation, adhesion, and development of cancer cells by blocking *miR-506*. Consequently, *NEAT1* and *LIN28B* together might be potent HGSOC biomarkers. In ovarian cancer cells, a high expression of *NEAT1* can increase basic leucine zipper and W2 domain-containing protein (BZW1) and inhibit *miR-4500*. *NEAT1* knockdown causes apoptosis and prevents the colony formation, migration, glycolysis, and proliferation of ovarian cancer cells [36].

*NEAT1* and *FGF9* are overexpressed in ovarian cancer cells. *NEAT1* targets *VEGF*, *Ang-1*, and *MMP2* to induce angiogenesis. The overexpression of *miR-365* or knockdown of *NEAT1* or *FGF9* reduces cell proliferation, colony formation, and angiogenesis. The effect of *FGF9* knockdown can be reversed by the overexpression of *NEAT1* or knockdown of *miR-365* [37]. In ovarian cancer tissues and cell lines resistant to paclitaxel (PTX), *NEAT1* also lowers miR-194. *ZEB1* expression is stimulated by *NEAT1* sponging *miR-194*, which also causes EMT and a drug-resistant phenotype. The knockdown of *NEAT1* increases PTX-induced apoptosis in a cohort of PTX-resistant ovarian cancer cells in vitro, accelerating cell drug sensitivity. Treatment resistance is also decreased by *NEAT1* knockdown in vivo [38]. *NEAT1* expression is upregulated and *let-7g* is downregulated in ovarian cancer cells. *Let-7g* is competitively bound by *NEAT1*, which lowers the expression of *let-7g*. *Let-7g* decreases the proliferation, migration, and invasion of cancer cells while also increasing the synthesis of adipose triglyceride lipase (ATGL) and inhibiting the expression of mesoderm-specific transcript (*MEST*). Conversely, *NEAT1* silencing inhibits the growth of xenograft tumors [39].

## 3. Long Non-Coding RNA SOX21-AS1

Chromosome *13q32.1* contains the LncRNA *SOX21* antisense RNA1 (*SOX21-AS1*), which is involved in the initiation of some malignancies. By epigenetically suppressing *p21* expression, *SOX21-AS1* is linked to the advancement of hepatocellular carcinoma and may be utilized to forecast the prognosis of patients [40].

The development and spread of cancer may be primarily caused by aberrant amounts of lncRNA. The long non-coding RNA *SOX21-AS1* has an up-stream role and could represent a novel oncogene in a number of cancers, such as melanoma, osteosarcoma, breast, lung, and ovarian cancer. Mostly functioning as a competitive endogenous RNA (*ceRNA*), *SRY-box* transcription factor 21 antisense divergent transcript 1 (*SOX21-AS1*) suppresses the amount of its target microRNAs (*miRNAs*), causing their targets to be upregulated. In addition, *SOX21-AS1* participates in the signaling pathways of transforming growth factor-β (*TGF-β*), *Wnt*, and phosphatidylinositol 3-kinase (*PI3K*)/protein kinase B (AKT). Affected patients’ clinicopathological characteristics and lncRNA are also connected. The overexpression of *SOX21-AS1* has been linked to processes connected to cancer, including cell cycle arrest, invasion, migration, apoptosis, and epithelial–mesenchymal transition (EMT). Given *SOX21-AS1’s* association with poor prognosis and shorter patient survival, it may be used as a prognostic biomarker and diagnostic biomarker in cancer. Furthermore, *SOX21-AS1* makes ovarian cancer cells more resistant to the chemotherapeutic drug cisplatin [41].

## 4. Long Non-Coding RNA H19

The first lncRNA that Brannan found was *H19*. This gene, which is paternally imprinted and situated close to the telomeric region of chromosome *11p15.5*, is frequently implicated in the development of tumors [42,43]. Although *H19* is re-expressed in tumor tissues, it is not expressed in postnatal tissues [44]. A number of malignancies, including ovarian, breast, stomach, and esophageal cancers, have been linked to disturbed *H19* levels [45,46,47]. LncRNA *H19* is believed to play an important role in the development of ovarian cancer by regulating various pathways, as evidenced by increased expression levels in cisplatin-resistant *A2780-DR* cells. Studies conducted in vitro and in vivo have demonstrated that *H19* knockdown results in a decreased expression of *NRF2*-targeted proteins like *G6PD*, *GSR*, *NQO1*, *GCLM*, *GSTP1*, and *GCLC* and the enhanced susceptibility of *A2780-DR* cells to cisplatin. H19 modifies glutathione metabolism, which also leads to treatment resistance [48]. *H19* is essential for the epithelial-to-mesenchymal transition (EMT) that is brought about by the release of spongiform *miR-370-3p* [49]. Warburg effect regulation is accomplished via *H19*’s alteration of glycolytic metabolism. By sponging *miR-324-5p*, *H19* silencing lowers lactate generation, *PKM2* expression, and glucose consumption [50].

In the ovarian cell line *A2780*/*CP*, which is resistant to cisplatin, valproic acid lowers *H19* expression. The *A2780*/*CP* cell line’s susceptibility to cisplatin and rate of apoptosis both significantly increase upon the subsequent silencing of *H19* [51]. On the other hand, *H19* expression was shown to be considerably higher in the cisplatin-resistant ovarian cancer cell line *OVCAR3*/*DDP* than in the *OVCAR3* cell line. Slug, twist, and snail were among the EMT markers that *OVCAR3*/*DDP* cells overexpressed, while E-cadherin was downregulated. In *OVCAR3*/*DDP* cells, subsequent *H19* knockdown resulted in the promotion of E-cadherin expression and the inhibition of migration and EMT-positive markers [52]. Ginsenoside *Rg3* inhibits OC cell proliferation, migration and invasion by partially inhibiting the expression of lncRNA *H19*. *H19* suppression was identified as the cause of the ginsenoside Rg3 treatment’s reduction in N-cadherin expression and increase in E-cadherin levels [53].

## 5. Long Non-Coding RNA Lysyl Oxidase-like 1 Antisense RNA 1 (LOXL1-AS1)

Lysyl oxidase-like 1 antisense RNA 1 (*LOXL1-AS1*) is am lncRNA found on human chromosome *15q24.1*. It plays a role in the development and progression of a number of cancers, including gastric cancer, bladder cancer, prostate cancer, ovarian cancer, cervical cancer, breast cancer, glioma, thymus cancer, liver cancer [54] and pancreatic cancer [55]. 

Patients with ovarian cancer exhibit a notably elevated expression level of *LOXL1-AS1* in comparison to healthy individuals. The expression level of *LOXL1-AS1* is associated with distant metastases and an advanced FIGO stage. Some findings suggested that patients with ovarian cancer who had higher levels of circulating *LOXL1-AS1* may have experienced a worse overall survival rate [56]. Since *LOXL1-AS1* expression in OC cells is substantially higher than in normal ovarian cells, downregulating it will cause OC cells to proliferate less rapidly and undergo apoptosis by specifically regulating *miR-761* [57]. By the *miR-18b-5p*/*VMA21* axis, *LOXL1-AS1* stimulates the proliferation, migration, and invasion of OC cells [58]. *LOXL1-AS1* is a putative target for OC therapy as it is a possibly carcinogenic lncRNA in OC. 

## 6. Long Non-Coding RNA MAFG-AS1

Chromosome 17 is the location of the transcription factor MAF BZIP G-antisense RNA 1 (*MAFG-AS1*). In addition to suppressing 16 miRNAs and directly influencing the expression of 22 protein-coding genes, *MAFG-AS1* is elevated in 15 human malignancies [59]. Tumor size, clinical stage, distant metastases, overall survival, and disease-free survival are all highly associated with *MAFG-AS1* levels. Through cell proliferation, invasion, glycolysis, metastasis, and drug sensitivity, *MAFG-AS1* contributes to the development of disease.

The mechanism underlying the interaction between *MAFG-AS1*, *miR-339-5p*, *NFKB1*, and *IGF1* was identified by characterizing their binding affinities by Bai et al. In order to examine the impact of *MAFG-AS1* overexpression or knockdown on OC cell invasion, migration, and EMT, they also silenced *NFKB1* and *IGF1*. Nude mice were used for xenografts in order to validate the in vitro findings. In OC tissues and cells, *MAFG-AS1* was shown to have a considerably high expression pattern. It was also shown that *MAFG-AS1* recruited *NFKB1* to enhance the expression pattern of *IGF1*, and that it bound to *miR-339-5p* to raise the expression pattern of *NFKB1*. Therefore, it can be said that suppressing *IGF1* or *NFKB1* would reverse the effects of *MAFG-AS1* overexpression, which increases the EMT, invasion, and migration of OC cells. Consequently, *MAFG-AS1* might offer an opportunity for treatment against OC [60,61].

## 7. Long Non-Coding RNA GAS5

LncRNA growth arrest specific 5 (GAS5) is the source of multiple non-coding short nucleolar RNAs (snoRNAs) [62]. Numerous malignancies have been shown to have altered *GAS5* levels. *GAS5* has the potential to control cisplatin resistance in cervical cancer by modulating microRNA-21 (*miR-21*) [63]. Reduced lncRNA *GAS5* levels in OC are frequently linked to a poor prognosis [64]; regrettably, further research is needed to understand the biological mechanism of *GAS5* in OC. The effect of altered *GAS5* on ovarian cancer cell phenotypes was investigated in vitro and in vivo. The results showed that *GAS5* is decreased in cancer tissues, especially in tumors with a larger size, deeper invasiveness and higher tumor stage. Patients with lower levels of *GAS5* expression had worse disease-free survival (*p* < 0.0001) and overall survival (*p* = 0.0016) than patients with high *GAS5* expression. In contrast, *GAS5* overexpression inhibits ovarian cancer cell proliferation in vitro and in vivo. *GAS5* influences ovarian cancer cell proliferation by regulating the expression of cyclin *D1*, *p21*, and apoptosis protease-activating factor 1 (*APAF1*). LncRNA *GAS5* may therefore be a potential indicator of poor prognosis in ovarian cancer and a therapeutic target [65].

By decreasing the expression of *miR-96-5p*, *GAS5* overexpression can drastically impair the ability of ovarian cancer cells to proliferate and invade. *GAS5* may also have an impact on the signaling pathway that involves PTEN, protein kinase B (AKT), and *mTOR* [66]. In ovarian cancer, a higher tumor volume and an FIGO stage (III–IV) are associated with low *GAS5* expression and high *miR-196a-5p* expression [67]. *GAS5* downregulation causes ovarian cancer cells to proliferate more quickly, experience a lower rate of apoptosis, and grow larger tumors in rats. *GAS5* has the ability to bind and control *miR-196a-5p* directly. *miR-196a-5p* expression is elevated in ovarian cancer tissues and cell lines, indicating that it may favor ovarian cancer [68]. In ovarian cancer cells, high expression levels of *GAS5* promote apoptosis. 

Ma et al. found that ovarian cancer tissues and cell lines exhibit significantly higher expression levels of microRNA (*miR)-21* than adjacent non-cancerous tissues and normal ovarian epithelial cells but significantly lower expression levels of *GAS5* and Sprouty homolog 2 (*SPRY2*). The luciferase experiment revealed that, in ovarian cancer-derived *A2780* cells, *miR-21* was directly targeted by *GAS5*, and that its target gene was *SPRY2*. The downregulation of *miR-21* and upregulation of *SPRY2* are observed in conjunction with a considerable inhibition of ovarian cancer cell growth caused by *GAS5* overexpression. On the other hand, *A2780* cell growth is significantly inhibited by *miR-21* overexpression, along with a decrease in *SPRY2* expression. Additionally, the overexpression of *miR-21* enhances *GAS5*’s stimulating influence on *SPRY2* expression while attenuating its suppressive effect on *A2780* cell proliferation. On the other hand, *SPRY2* knockdown restores *GAS5*’s inhibitory effect on *A2780* cell proliferation. Thus, by first suppressing the expression of *miR-21* and then upregulating the expression of *SPRY2*, *GAS5* inhibits the proliferation of ovarian cancer cells. Thus, the *GAS5*/*miR-21*/*SPRY2* signaling pathway could be a viable target for treatment in ovarian cancer [69].

The malignant tumor of the female reproductive system with the highest fatality rate is epithelial ovarian cancer (EOC). Tolerance to early-stage chemotherapeutic medications, such as cisplatin, is a significant factor contributing to a poor prognosis in EOC. Long et al. investigated the in vitro and in vivo effects of lncRNA GAS5 on human ovarian cancer cell lines *HEY*, *A2780*, *A2780*/*DDP*, *HO8910*, *HO8910PM*, *SKOV3*, and *SKOV3*/*DDP* and a normal human ovarian epithelial cell line. They found an extremely low expression of lncRNA *GAS5* in epithelial ovarian cancer (EOC) samples, which was associated with prognosis. While *GAS5* overexpression greatly improved the sensitivity of OC cells to cisplatin in vivo and in vitro, it also caused *G0*/*G1* OC cell arrest and increased apoptosis. Low *GAS5* expression was also seen in cisplatin-resistant OC cell lines. By attracting the *E2F4* transcription factor to the promoter of *PARP1*, *GAS5* may control the transcription factor’s activity, which in turn affects the *MAPK* pathway’s activity. Because of the 5’TOP structure, the transcription inhibitor rapamycin can control *GAS5* in OC cells [70].

## 8. Long Non-Coding RNA LINC00152

Long intergenic non-coding RNA 00152 (*lncRNA LINC00152*) is an *828-bp* lncRNA located on chromosome *2p11.2*. Many malignancies, including colorectal, liver, gastric, breast, ovarian, lung, pleural, and glioblastoma, express it aberrantly [71,72,73,74].

When compared to normal tissue, epithelial ovarian cancer tissue exhibits a considerable increase in *LINC00152* expression. In *SKOV3* cells, *LINC00152* controls both cell cycle and proliferation [75]. *LINC00152* mediates cell proliferation, thereby affecting *MCL-1* expression and mitochondrial apoptosis pathways via MCL-1, and acts as a competitive endogenous RNA (*ceRNA*) of *miR-125b*, which may represent a novel molecular mechanism for reversing cell proliferation in ovarian cancer [76]. Furthermore, the cisplatin sensitivity of epithelial ovarian cancer cells is enhanced by *LINC00152* knockdown, which lowers *MDR1*, *MRP1*, and *GST* expression levels and enhances apoptosis [77]. Additionally, it is thought that *LINC00152* can attach to B-cell lymphoma 6 (BCL6)’s *Ser333*/*Ser343* and stabilize it against ubiquitination to encourage the growth and invasion of ovarian tumors [78].

## 9. Pseudogenes

Pseudogenes, or faulty copies of genes produced during genome evolution, are referred to as “junk DNA” [79]. Only two-thirds of the nearly 18,000 pseudogenes found in the human genome are transcribed [80]. It is possible for pseudogenes to control both transcriptional and post-transcriptional aspects of gene expression. In ovarian cancer, the dysregulation of many pseudogenes has been reported [81]. Nuclear proteins known as high-mobility group A (HMGA) aid in the construction of nucleoprotein complexes related to transcription, gene replication, and chromatin architecture. Proto-oncogenic competitive endogenous RNAs are *HMGA1P6* and *HMGA1P7* [82]. High-mobility group AT-hook 1 pseudogene 6, or *HMGA1P6*, is overexpressed in HGSOC and exhibits an inverse relationship with patient survival. By functioning as a competitive endogenous RNA (ceRNA) and causing an increase in the production of *HMGA1* and *HMGA2*, *HMGA1P6* mechanically increases the malignancy of ovarian cancer cells. In ovarian cancer, the *MYC* oncogene transcriptionally activates *HMGA1P6* [83]. The overexpression of *HMGA1*/*2* is found to be inversely associated with miRNAs [84].

## 10. Circular RNAs

Circular RNAs (CircRNAs) are single-stranded RNAs covalently linked at the 5′ and 3′ ends that are generated by back-splicing from pre-mRNA [85]. CircRNAs most often act through sponging microRNAs (miRNAs) to influence target gene expression. They also interact with proteins to modulate their activity and regulate host gene transcription [86,87,88]. The dysregulation of circRNA causes the development of many diseases, especially cancer [89]. Many circRNAs are dysregulated in EOC and can be used as diagnostic and prognostic markers. We described selected circRNAs dysregulated in OC in Table 1, focusing on their level in OC and clinical significance.

## 11. Treatment of Ovarian Cancer with lncRNA-Modulating Drugs

A clinical trial focuses on the innovative combination of two targeted inhibitors, Palbociclib and Bevacizumab, using long non-coding RNAs (lncRNA) as biomarkers for colorectal, lung, breast, and ovarian cancers. Palbociclib is a cyclin-dependent kinase 4/6 (CDK4/6) inhibitor that disrupts cell cycle progression, and Bevacizumab, on the other hand, is an inhibitor of vascular endothelial growth factor (VEGF), which promotes angiogenesis. The mechanisms of both drugs complement each other, and lncRNAs are used in predicting treatment response and prognosis [102]. LncRNA CTD-2589M5 is coexpressed with most multidrug resistance genes, namely ABCB1, ABCB4, ABCC3, and ABCG2, showing a role in multidrug resistance in ovarian cancer [103]. Information about the mechanisms of action and clinical significance of lncRNAs is listed in Table 2.

## 12. Conclusions

1. Some LncRNAs such as lncRNA NEAT1 and SOX21-AS1 can be used in the diagnosis of OC; however, further research and standardization are necessary.
2. LncRNA SOx21-AS1, NEAT1 and GAS5 can also be used as indicators of poor prognosis in OC, but further research and standardization are also necessary.
3. Treatment with lncRNA H19, MAFG-AS1 or GAS5 is a promising treatment for ovarian cancer, although further research is needed in this area. Unfortunately, the heterogeneity of epithelial ovarian cancer may constitute a limitation in the development of an effective treatment method using lncRNA.
4. Resistance to drugs such as cisplatin remains the main problem in the treatment of OC. Although HGSOC is at least initially susceptible to platinum-based drugs, this is not the case for low-grade serous ovarian cancers or clear cell ovarian cancers. LncRNA NEAT1 and H19 may prove helpful in the fight against chemotherapy resistance, but further research is necessary.

A limitation of the study may be the fact that most studies refer to cell lines. The profile and activity of different lncRNAs differ significantly; therefore, it is necessary to better understand the mechanisms of action of each lncRNA.

## Figures and Tables

**Table 1:** Selected dysregulated circRNAs in OC.

circRNA	Level in OC	Clinical Use	Reference
circHIPK3	Up	Higher circHIPK3 expression is associated with lymph node invasion, the FIGO stage and a worse value of the DFS and OS of patients.	[90]
circFAM120A (circ_0003972), circTOM1L1 (circ_0007288)	Down	They can serve as biomarkers in the diagnosis of OC.	[91]
circN4BP2L2	Down	It may serve as a promising new diagnostic biomarker for patients with EOC.	[92]
circBNC2 (circ_0008732)	Down	It inhibits the progression of OC by regulating the miR-223-3p/FBXW7 axis, so it may be a potential biomarker in OC therapy.	[93]
circPLEKHM3 (circ_0001095)	Down	It acts as a tumor suppressor in OC cells by targeting the miR-9/BRCA1/DNAJB6/KLF4/AKT1 axis, so it can be used as a prognostic indicator and therapeutic target in OC patients.	[94]
circFBXO7 (circ_0001222)	Down	It acts as a tumor suppressor in OC. The circFBXO7/miR-96-5p/MTSS1 axis is an important regulator of the Wnt/β-catenin signaling pathway, which may be a promising target in OC therapy.	[95]
circRNA1656 (circ_0002755)	Down	It may serve as a new tumor marker for HGSOC.	[96]
circSMARCA5 (circ_0001445)	Down	It has an antigenic effect by targeting the miR-576-5p/SFRP1 axis and blocking the progression and development of OC. It can therefore be used as an indicator or possible therapeutic target in OC patients.	[97]
circRHOBTB3 (circ_0007444)	Down	It is a neoteric biomarker and a promising target of EOC.	[98]
circSLC22A3 (circ_0078607)	Down	It may serve as a sponge for miR-32-5p to regulate SIK1 expression, thereby inhibiting the progression of OC.	[99]
circMYLK	Up	Its expression is associated with the pathological stage and poor prognosis in patients with OC.	[100]
circABCB10	Up	It correlates with advanced clinicopathological features and unfavorable survival and promotes proliferation and reduces apoptosis.	[101]

DFS—disease-free survival, OS—overall survival, OC—ovarian cancer, EOC—epithelial ovarian cancer, HGSOC—high-grade serous ovarian cancer.

**Table 2:** Summary of information on selected long non-coding RNAs in ovarian cancer.

LncRNA	The Most Important Mechanisms of Action	Clinical Aspect
NEAT1	- mediates the expression of tight junction protein three (TJP3) and blocks the activity of miR-1321 [30] - binds to the human antigen R (HuR) protein and spongy miR-124-3p [32] - blocks the action of caspase-3 [34] - accelerates the cell’s entry into the S phase and shortens the G0/G1 phase [34] - blocks miR-506 [36] - a high expression of NEAT1 can increase the basic leucine zipper and W2 domain-containing protein (BZW1) and inhibit miR-4500 [36] - induces angiogenesis by affecting VEGF, Ang-1 and MMP2 [37] - downregulates miR-194 in ovarian cancer tissues and paclitaxel (PTX)-resistant cell lines [38]	Abnormal expression is associated with metastasis, treatment resistance, and patient survival [27,28].
SOX21-AS1	- acting as a competitive endogenous RNA (ceRNA), antisense divergent transcript 1 of SRY-box transcription factor 21 (SOX21-AS1) represses the amount of target microRNAs (miRNAs) [41] - participates in the transforming growth factor β (TGF-β), Wnt and phosphatidylinositol 3-kinase (PI3K)/protein kinase B (AKT) signaling pathways [41]	It can be used as a prognostic biomarker and diagnostic biomarker in OC [41].
H19	- H19 knockdown results in a decreased expression of NRF2-targeted proteins such as G6PD, GSR, NQO1, GCLM, GSTP1, and GCLC and an increased susceptibility of A2780-DR cells to cisplatin [48] - modifies glutathione metabolism [48]	It plays an important role in creating resistance to cisplatin in the treatment of OC, which may be an important starting point in therapy [48].
LOXL1-AS1	- stimulates OC cell proliferation, migration and invasion through the miR-18b-5p/VMA21 axis [58]	It is the putative target of OC therapy [58].
MAFG-AS1	- interacts with miR-339-5p, NFKB1 and IGF1 through binding affinity [60,61]	It is a potential target in the treatment of OC [60,61].
GAS5	- regulates the expression of cyclin D1, p21 and apoptosis protease-activating factor 1 (APAF1) [65] - reduces miR-96-5p expression [66] - affects signaling pathways including PTEN, protein kinase B (AKT) and mTOR [66] - controls miR-196a-5p [68] - is involved in the GAS5/miR-21/SPRY2 pathway [69]	It is a potential indicator of poor prognosis and a therapeutic target in OC [65,69].
LINC00152	- controls cell cycle and proliferation in SKOV3 cells [75] - affects MCL-1 expression and mitochondrial apoptosis pathways through MCL-1 and acts as a competitive endogenous RNA (ceRNA) for miR-125b [76] - LINC00152 knockdown reduces the expression levels of MDR1, MRP1 and GST and increases apoptosis [77]	It plays an important role in creating resistance to cisplatin in the treatment of OC [77].

NEAT1—nuclear-enriched autosomal transcript 1, SOX21-AS1—SOX21 antisense RNA1, LOXL1-AS1—Lysyl oxidase-like 1 antisense RNA 1, MAFG-AS1—factor MAF BZIP G-antisense RNA 1, GAS5—growth arrest-specific 5, LINC00152—long intergenic non-coding RNA 00152, OC—ovarian cancer.
